# Supplementary material for: Ultrabright gap-enhanced Raman tags for high-speed bioimaging
Source: Nat Commun. 2019 Aug 29;10:3905. doi: 10.1038/s41467-019-11829-y (PMC6715656; doi:10.1038/s41467-019-11829-y)
Supplement: Supplementary file 1 — Supplementary Information [file 41467_2019_11829_MOESM1_ESM.pdf]

Supplementary Information

Ultrabright gap-enhanced Raman tags for high-speed bioimaging

Yuqing Zhang et al.

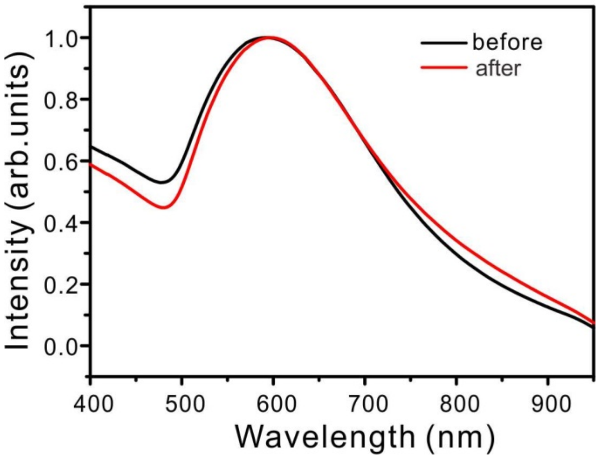

Supplementary Figure 1. Normalized extinction spectra of P-GERTs before and after the absorption of 4-NBT reporters on the surface of the external shell.

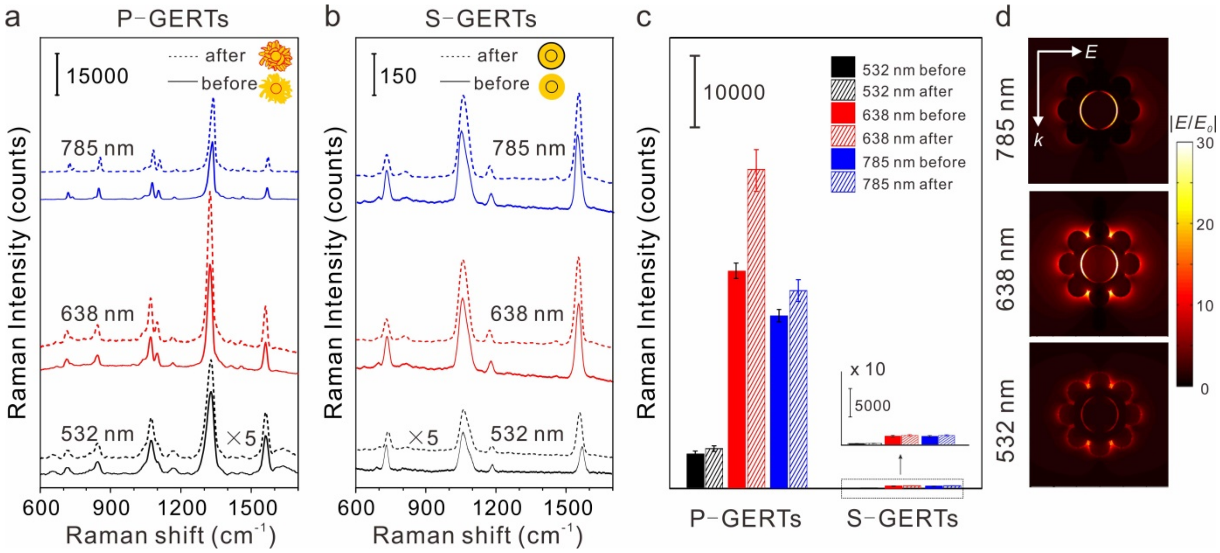

Supplementary Figure 2. Excitation wavelength dependent Raman spectra of P-GERTs and S-GERTs. Raman spectra of (a) P-GERTs and (b) S-GERTs before and after adsorption of reporter molecules on the external surface of the shells excited by 532, 638 and 785 nm laser. (c) Comparison of Raman band intensity (1340 cm^{-1} for P-GERTs and 1055 cm^{-1} for S-GERTs) of all spectra in panel A and B. (d) FDTD calculated electric field enhancement distribution of a single P-GERT at excitation wavelengths of 532, 638 and 785 nm. Source data are provided as a Source Data file.

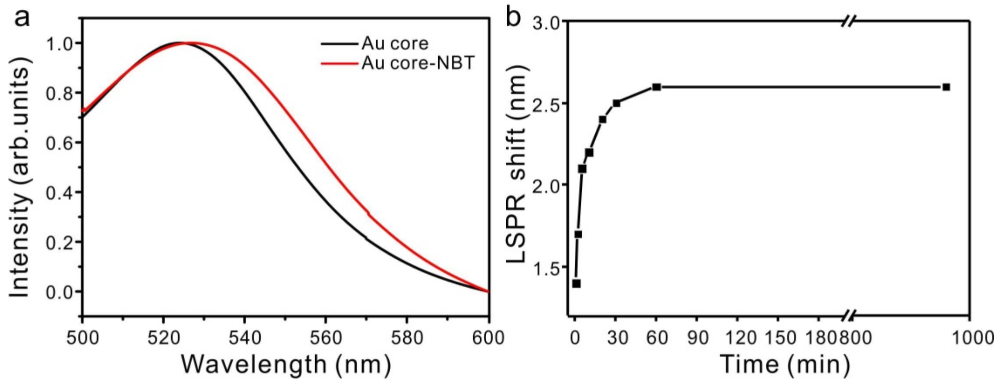

Supplementary Figure 3. (a) Normalized extinction spectra of intact Au cores and Au cores incubated with 4-NBT molecules for 960 min. (b) LSPR shifts of the mixture of Au cores and 4-NBT molecules during 960 min incubation.

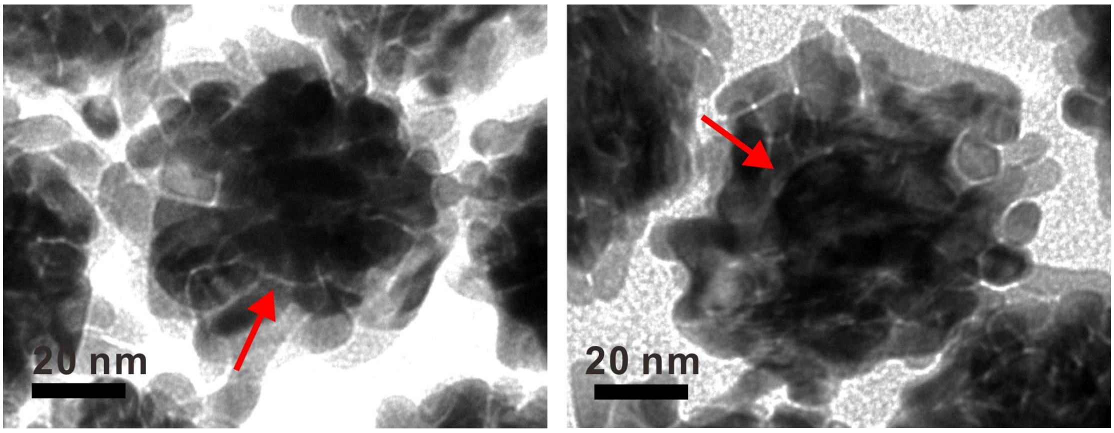

Supplementary Figure 4. More representative TEM images of P-GERTs when the incubation time of Au cores and 4-NBT molecules is 960 min. All red arrows indicate the internal nanogaps. The scale bars are 20 nm. Source data are provided as a Source Data file.

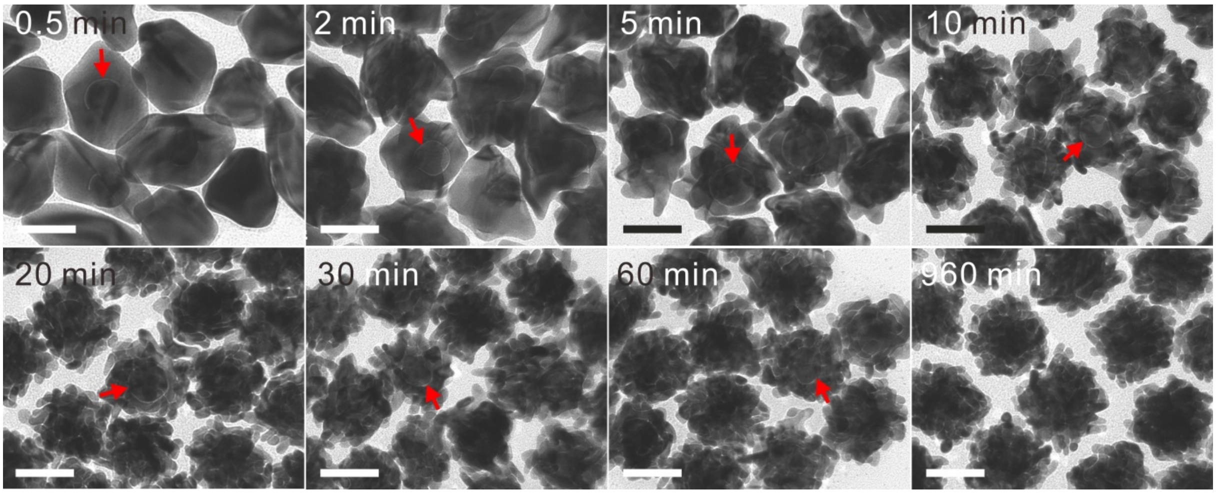

Supplementary Figure 5. Representative TEM images of P-GERTs as a function of incubation time (0.5, 2, 5, 10, 20, 30, 60, and 960 min). All scale bars are 50 nm. All red arrows indicate the internal nanogaps of P-GERTs.

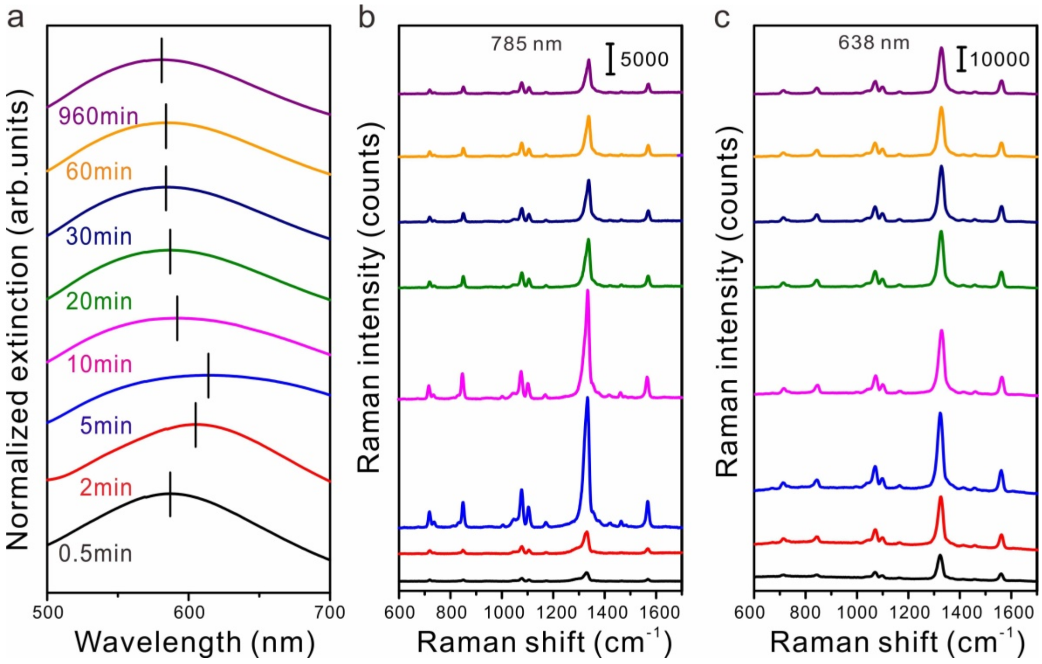

Supplementary Figure 6. (a) Extinction spectra and Raman spectra of aqueous P-GERTs as a function of incubation time (bottom to top: 0.5, 2, 5, 10, 20, 30, 60, and 960 min) excited by (b) 785 nm and (c) 638 nm laser.

Supplementary Note 1: Far- and near-field optical properties of P-GERTs with different morpholgies. The extinction spectra of P-GERTs with eight different incubation time are shown in Supplementary Figure 6a, all exhibiting only one plasmon resonance peak in the

visible range. As the incubation time is increased from 0.5 to 5 min, the resonance peak gradually redshifts from 584 to 610 nm, accompanied by a significant broadening of the resonance linewidth. However, as the incubation time is further increased from 5 to 960 min, the resonance peak gradually blueshifts from 610 to 574 nm, accompanied by a slight linewidth narrowing. The near-field optical properties of P-GERTs with varying incubation time are investigated by measuring the averaged SERS signals. We firstly investigate the Raman enhancement contribution from the internal near-field hot spots with the influence by the Au shell morphology induced by the incubation time. The Raman spectra and the band intensities at 1340 cm^{-1} of P-GERTs (without the external decoration of 4-NBT) with different incubation time for the excitation of 638 and 785 nm were plotted for comparison in Supplementary Figure 6b. They all exhibit a similar trend that the Raman intensity increases at the initial stage and reaches the maximum when the incubation time is 5 min but then decreases when the incubation was further prolonged until to 960 min. Both this phenomenon and the aforementioned far-field extinction spectral evolution can be explained by the morphology changes of P-GERTs. The morphology changes of P-GERTs may related to the adsorption amount of 4-NBT molecules on Au cores and the poor affinity between Au and the nitro group. For the reason that the Raman signal intensity decreases as the incubation time increases from 5 to 960 min, we presume that the internal nanogaps become more incomplete during this process, which also leads to the much weaker Raman enhancement.

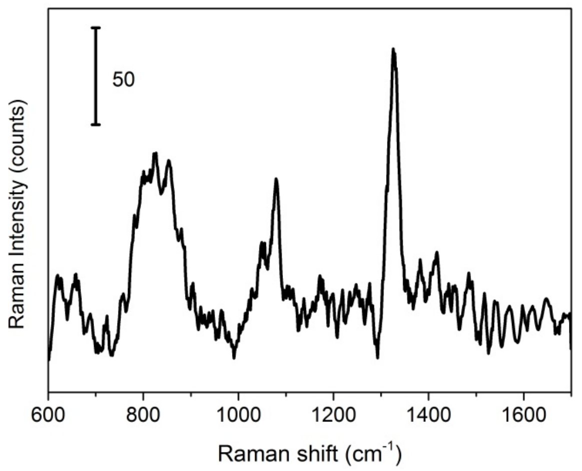

Supplementary Figure 7. Raman spectra of aqueous P-GERTs with a concentration of 1 fM (638 nm laser, 20 mW, 10 s acquisition time, and 60× objective lens).

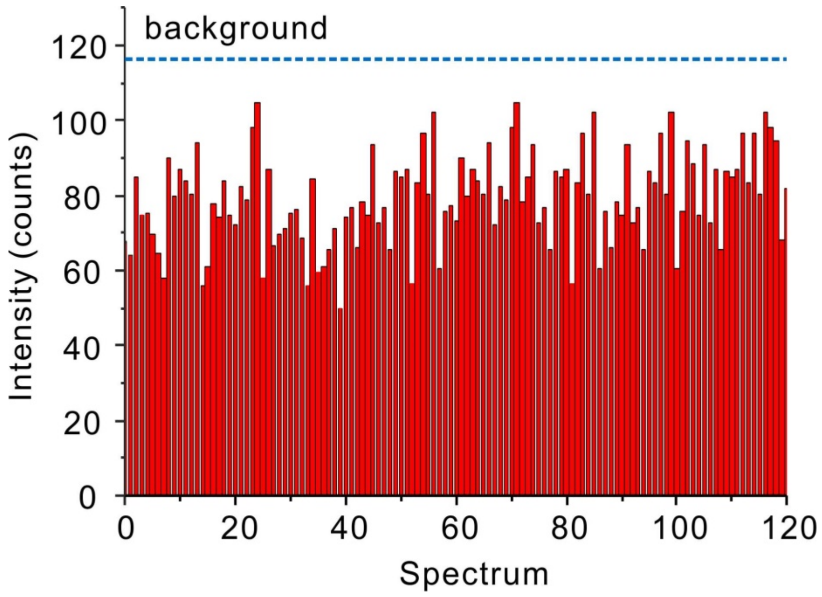

Supplementary Figure 8. Background Raman signals (1340 cm^{-1}) measured for 120 spectra from the solution without P-GERTs. The blue dot line represents the threshold intensity of 117 counts.

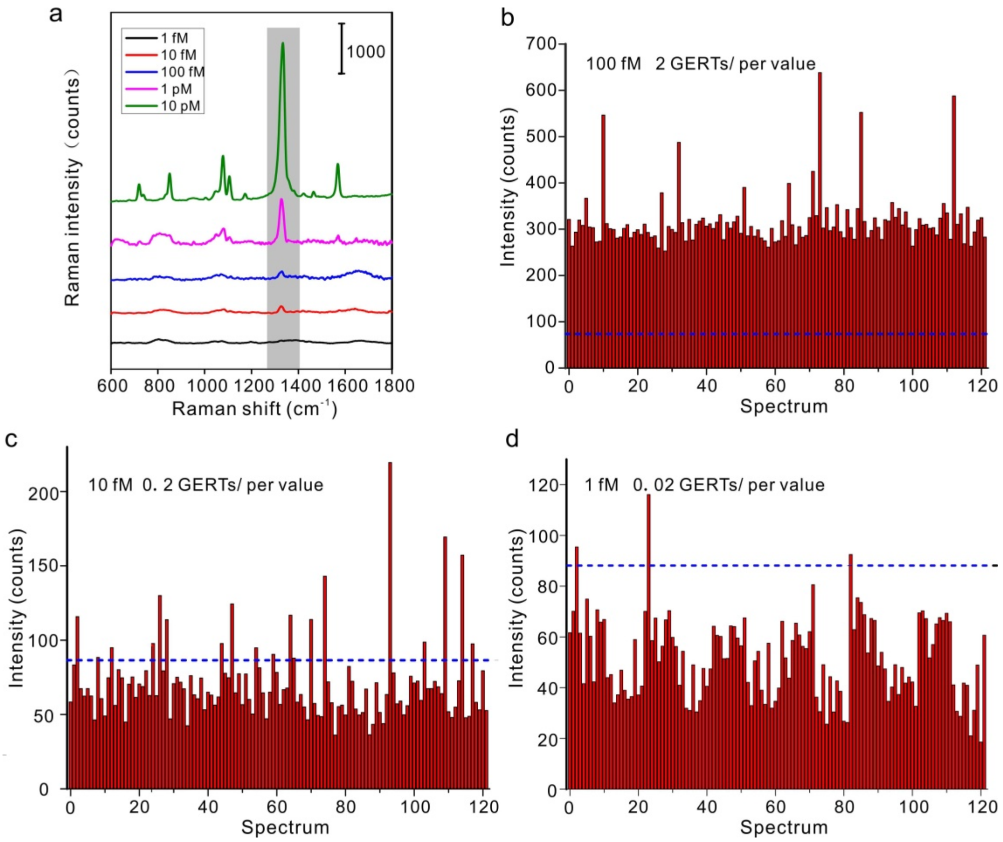

Supplementary Figure 9. Single-NP detection of aqueous MS P-GERT solution at 785 nm. (a) Concentration-dependent Raman measurements of aqueous P-GERTs (785 nm laser, 20 mW, 10 s acquisition time, and 60× objective lens). The intensity of Raman band at 1340 cm^{-1} of 120 measurements from (b) 100, (c) 10, and (d) 1 fM P-GERT solution. 100, 10, and 1 fM GERT solutions correspond to 2, 0.2, and 0.02 particle per probing volume, respectively. Blue dotted lines in panel (b-d) represent the background Raman signal (1340 cm^{-1}) without P-GERTs. The blue dot lines present the threshold of the background Raman signal.

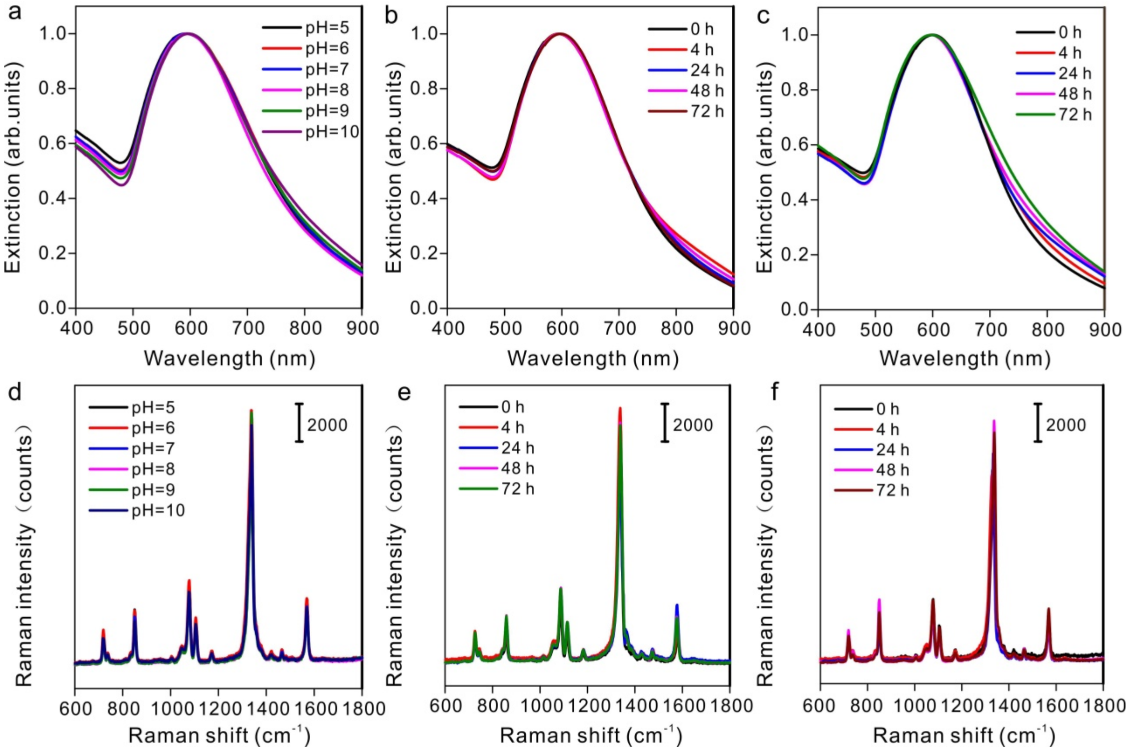

Supplementary Figure 10. Extinction spectra of MS P-GERTs in (a) aqueous solutions of different pH values in the range of 5–10, (b) 10% FBS and (c) saline for 72 h. Raman spectra of MS P-GERTs in (d) aqueous solutions of different pH values in the range of 5–10, (e) 10% FBS and (f) saline for 72 h.

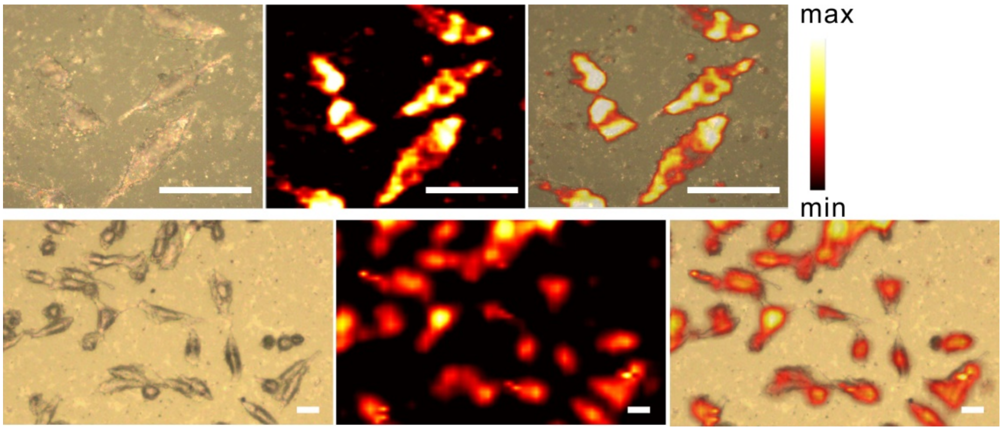

Supplementary Figure 11. Large-area SERS images containing multiple H1299 cells. The top Raman image (110 × 140 μm^2, 50 × 50 pixels) was obtained within 6 s, and the bottom Raman image (380 × 240 μm^2, 61 × 41 pixels) was obtained within 7 s. All scale bars are 50 μm. The Raman images are plotted using the Raman band (1340 cm^{-1}) of 4-NBT.

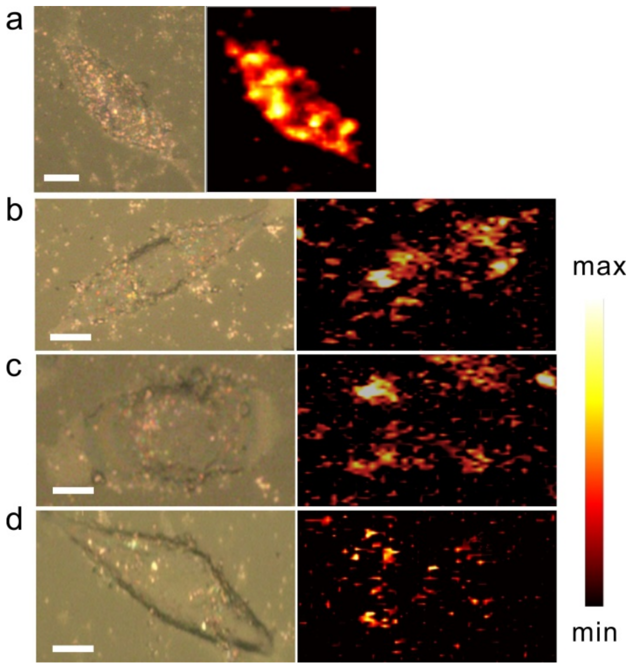

Supplementary Figure 12. Representative SERS imaging of cells containing different numbers of P-GERTs: bright-field images (left) and Raman images of different signal intensities labelled with (a) ~5000, (b) ~1000, (c) ~450 and (d) ~80 P-GERTs. Scale bar is 10 μm. All Raman images are plotted using the Raman band (1340 cm^{-1}) of 4-NBT with a resolution of ~2500 pixels.

Supplementary Note 2: Estimation of the number of intracellular P-GERTs. Based on the results of single nanoparticle nano-Raman analysis, we estimated that the signal intensity of the single P-GERTs was about 10 counts under the condition of ultrafast cell imaging (0.7 ms, 3.6 mW). Then we selected cell Raman images with different signal intensities (strong, medium, and weak) and estimated the number of labelled P-GERTs. The cell Raman image (as shown in above Supplementary Figure 12a) with excellent signals that can completely show the morphology of the cell contains about 5,000 particles. Cells Raman images (as shown in above Supplementary Figure 12b and c) with medium signals, which can basically show the outline of the cells, contains ~500 - 1000 particles. The cell Raman image (as shown in above Supplementary Figure 12d) with weak signals that can only show a part of the cell contains ~80 particles.

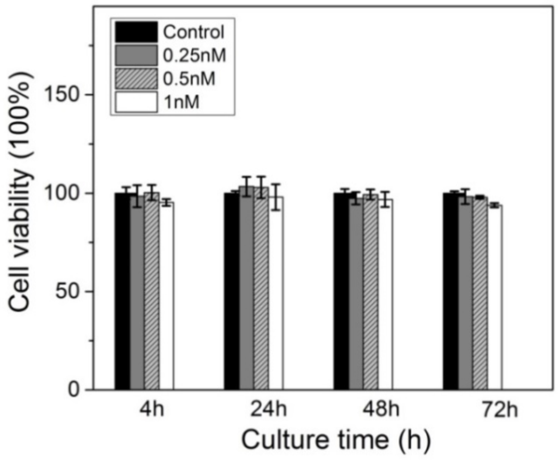

Supplementary Figure 13. Cellular viability of H1299 cells incubated with P-GERTs at different concentrations for 4, 24, 48, and 72 h. Data are shown as means ± SD.

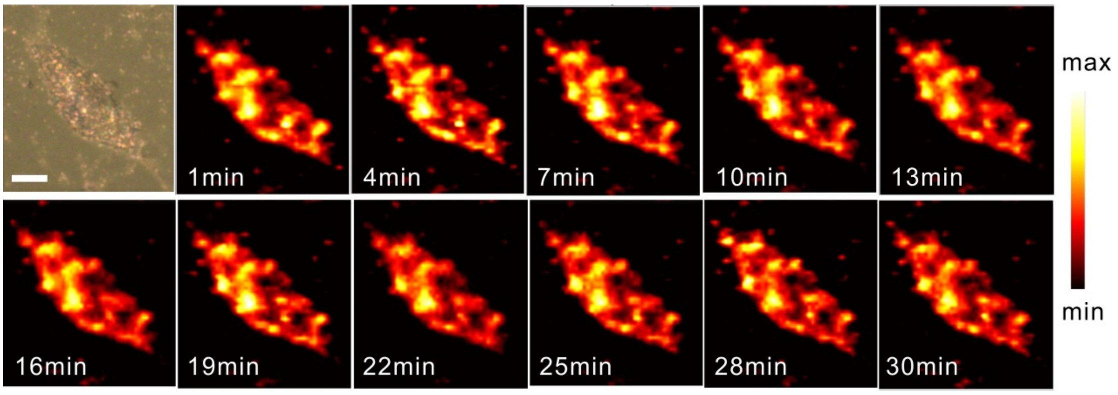

Supplementary Figure 14. Representative super-stable Raman images obtained from a single cell: bright-field image (top left) and Raman images at different irradiation times. Each Raman image (49 × 53 μm^2, 2500 pixels) was obtained within 6 s. Scale bar is 10 μm. The Raman image is plotted using the Raman band (1340 cm^{-1}) of 4-NBT.

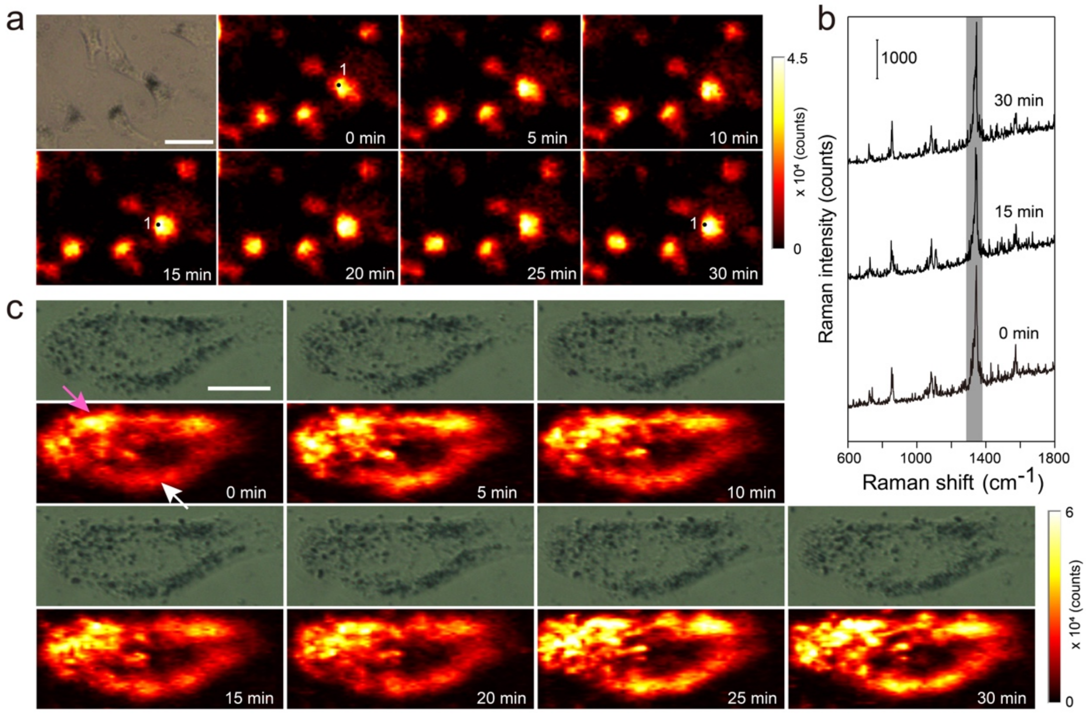

Supplementary Figure 15. Time-lapse live cell Raman images obtained from (a) multiple cells (135 × 180 μm^2, 2500 pixels) and (c) a single cell (80 × 32 μm^2, 2500 pixels). (b) Three representative Raman spectra at the point 1 (indicated in panel a) before, in the middle, and after the 30-min imaging process. Scale bar is 50 and 20 μm in panel a and c, respectively. All Raman image are plotted using the Raman band (1340 cm^{-1}) of 4-NBT.

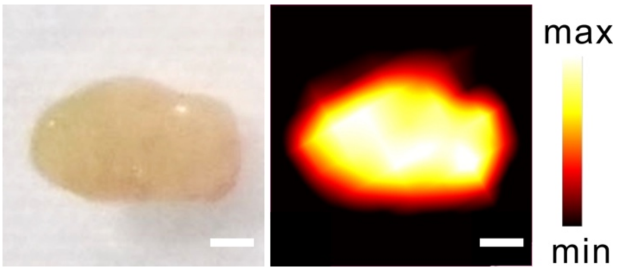

Supplementary Figure 16. A wide-area Raman image (right, 600 × 600 μm^2, 2500 pixels) of a dissected lymph node (left) obtained within 7 s under the DuoScan mode. All scale bars are 100 μm. The Raman image is plotted using the Raman band (1340 cm^{-1}) of 4-NBT.

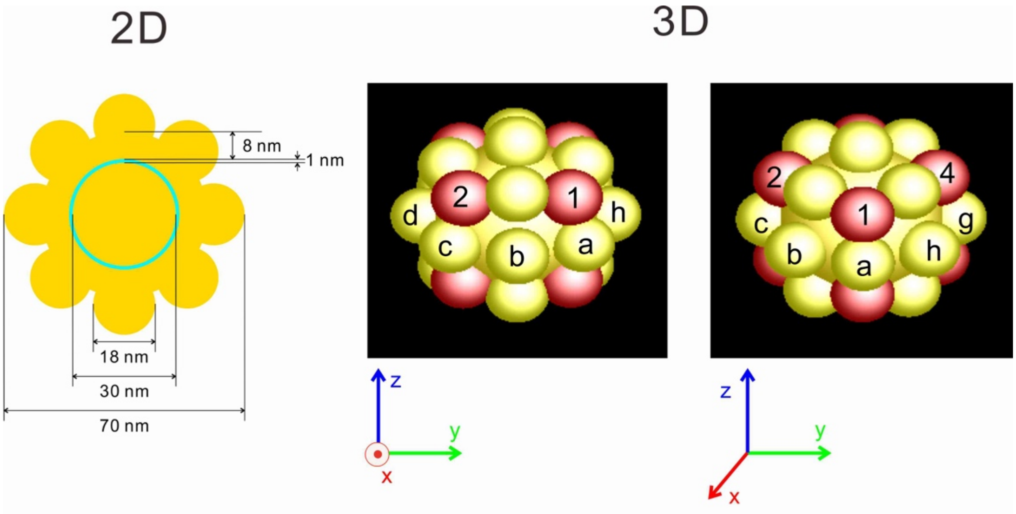

Supplementary Figure 17. The 3D model of a P-GERT used in FDTD simulation.

Supplementary Note 3: The 3D model of a P-GERT used in FDTD simulation**.** The real structure of P-GERTs in very complex with random petal-like shell structures and therefore we used some small Au nanospheres on the surface to mimic the petal-like structures. The P-GERT has an inner Au core of 30 nm in diameter, a gap of 1 nm in thickness, and a Au shell of 8 nm in thickness. The Au nanospheres on the surface are 18 nm in diameter and the total particle size is 70 nm. The total eighteen Au nanospheres (indicated by yellow color and with alphabetic letters, on the right of above Figure R6) were evenly distributed on three perpendicular circumferences of the particle along the *X*, *Y*, and *Z* axis, with eight nanospheres distributed on each direction. A 2 nm nanogap between the nanospheres is formed on the surface, which mimics the external nanogaps on the P-GERTs surface. Next, eight Au nanospheres (indicated by red color and with numbers, on the right of above Figure R6) are placed in the center of each rest blank areas. The distance between the center of red Au nanospheres and the center of the inner core is 26 nm. The red Au nanospheres may overlap with neighbor yellow Au nanospheres, which also tries to mimic the random petal-like structures.

Supplementary Note 4: Raman intensity comparison between aqueous P-GERTs and DNA-bridged SERS tags**.**[1] For DNA-bridged SERS tags, the Raman intensity is ~550 counts

with 633 nm laser, 10 s exposure time, 300 μW laser power and 0.5 nM concentration (see Figure 4 in Nature Nanotechnology, 2011, 6, 452-460). For P-GERTs, the Raman intensity is ~30000 counts with 633 nm laser, 2 s exposure time, 15 mW laser power and 0.2 nM concentration (see Figure 1 in our manuscript). After normalization of laser power, exposure time and particle concentration, the Raman intensity of P-GERTs (5000 counts/s·mW·nM) is more than one order larger than that of Au-NNPs (~370 counts/s·mW·nM).

Supplementary References

1. Lim, D. K. et al. Highly uniform and reproducible surface-enhanced raman scattering from DNA-tailorable nanoparticles with 1-nm interior gap. *Nat. Nanotechnol.* **6**, 452-460 (2011).
